# Cell-Mediated Immunity in Elite Controllers Naturally Controlling HIV Viral Load

**DOI:** 10.3389/fimmu.2013.00086

**Published:** 2013-04-09

**Authors:** Luca Genovese, Manuela Nebuloni, Massimo Alfano

**Affiliations:** ^1^AIDS Immunopathogenesis Unit, Division of Immunology, Transplantation and Infectious Diseases, San Raffaele Scientific InstituteMilan, Italy; ^2^Pathology Unit, L. Sacco Department of Biomedical and Clinical Sciences, University of MilanMilan, Italy

**Keywords:** HIV, elite controllers, long-term non progressors, AIDS, innate immunity, cytokines/chemokines

## Abstract

The natural course of human immunodeficiency virus (HIV) infection is characterized by high viral load, depletion of immune cells, and immunodeficiency, ultimately leading to acquired immunodeficiency syndrome phase and the occurrence of opportunistic infections and diseases. Since the discovery of HIV in the early 1980s a naturally selected population of infected individuals has been emerged in the last years, characterized by being infected for many years, with viremia constantly below detectable level and poor depletion of immune cells. These individuals are classified as “elite controllers (EC) or suppressors” and do not develop disease in the absence of anti-retroviral therapy. Unveiling host factors and immune responses responsible for the elite status will likely provide clues for the design of therapeutic vaccines and functional cures. Scope of this review was to examine and discuss differences of the cell-mediated immune responses between HIV+ individuals with disease progression and EC.

## Background

Hallmarks of human immunodeficiency virus (HIV) infection are the persistent depletion of immune cells, immune dysfunction, and inflammation, favoring the occurring of opportunistic infections and illnesses thus disease progression (Douek et al., 2003; Silvestri et al., 2003). In the natural course of HIV infection infected individuals are characterized by flu-like symptoms, rapid decrease of CD4+ T cells, and high viral loads (acute phase of infection, lasting 2–6 weeks), followed by stabilization of the number of CD4+ T cells and viral load (chronic phase of infection, lasting years). However, during the chronic phase a slow but constant decrease of the number of CD4+ T cells and increase of viral load occur. These events are further complicated by the chronic activation of the immune system, and inflammatory responses, favoring the occurring of co-morbidities. Arising of opportunistic infections and diseases is typical of the Acquired ImmunoDeficiency Syndrome (AIDS) phase, as defined when CD4+ T cell counts drop to <200 cells/μl of blood.

Over than the above described HIV infected individuals, defined as natural progressors, a restricted set of HIV infected individuals control infection naturally in the absence of anti-retroviral therapies. Two main populations have been described based either on their maintenance of peripheral CD4+ T cell counts for several years (long-term non-progressors, LTNP), or on their total or partial control of HIV viremia (elite controllers, EC).

Long-term non-progressors are characterized by being infected by more than 7 years, have stable CD4 T cell counts always above 500 cell/μl of blood and stable low but detectable viral loads, representing approximately 5% of all chronically HIV infected individuals.

Elite controllers represents a further restricted population (about 3/1000 of HIV infected individuals) (Deeks and Walker, 2007; Okulicz and Lambotte, 2011), and are defined by stable CD4 cell count (irrespective of a threshold), anti-retroviral therapy naïve, and with viral loads persistently below 50 copies/ml (undetectable) for more than 12 months (Okulicz and Lambotte, 2011).

Moreover, some individuals showed both features of LTNP and control of viral load, and have been defined as “elite LTNP” (for example, >8 years of HIV infection, CD4 cell nadir ≥600 μ with a positive slope of CD4 T cell counts) (Grabar et al., 2009). Indeed, in most cases, these cohorts of HIV-1+ individuals controlling disease progression only partially overlap, underlying different determinants of resistance to HIV-1 disease progression.

Elite controllers are also characterized by very low levels of viral load in the cerebrospinal fluid (below 2.5 copies/ml), whereas number of leukocytes and levels of inflammatory markers, such as albumin, neopterin, CCL2/MCP-1, and interleukin (IL)-10, overlap with those of uninfected or highly active anti-retroviral therapy (HAART)-treated HIV individuals (Probasco et al., 2010). Therefore, EC have the ability to control viral load both at the systemic level and in the central nervous system (CNS). These findings implicate that the virus either does not reach the CNS or that local immune responses control viral replication, although the lack of local inflammatory events suggest that a reduced amount of virus or replication occurred in CNS.

Indeed, control of viral loads in EC is temporary (Bailey et al., 2007; Goujard et al., 2009; Okulicz et al., 2009), and a durable control of viremia for more than 10 years occurs only in 1% of the individuals ascribed to this set of people (Lambotte et al., 2005; Grabar et al., 2009; Okulicz et al., 2009). Of particular interest is that AIDS events have been described also in few EC experiencing drop in CD4+ T cell count despite maintaining undetectable viral load (Pereyra et al., 2008, 2009; Sajadi et al., 2009). Studying transition from the EC status to the AIDS phase will provide invaluable insights for deeper comprehension of immune mechanisms controlling HIV infection and disease progression, thus for design of alternative or complementary therapies than HAART.

Therefore, EC represents a unique opportunity for unraveling if and how immune responses are controlling HIV infection. Indeed, it can be argued that individuals naturally restricting infection have been in contact with a deficient virus. However, viruses isolated from EC have replicative capacity as much as laboratory adapted strains (Migueles et al., 2003; Bailey et al., 2006b; Blankson et al., 2007; Lamine et al., 2007), no main viral genetic defects, or viral polymorphisms are responsible for the spontaneous control of HIV replication in these persons (Blankson et al., 2007; Miura et al., 2008), and synonymous substitutions occurs in *gag*, *pol*, and negative regulatory factor (*nef)* genes as a result of low level ongoing replication (Miura et al., 2009c; O’Connell et al., 2010; Salgado et al., 2010). GB virus C has been demonstrated to inhibit HIV replication in *in vitro* infected cells (Xiang et al., 2004; Jung et al., 2005) and in HIV+ individuals persistent co-infection with GBC virus is associated with prolonged survival (Zhang et al., 2006); however, it has been reported that frequency of GB virus C viremia was not different between EC and HIV progressors, thus not explaining the non-progressive course of disease in this elite cohort (Blankson et al., 2008).

Moreover, recent study has shown that the virus could be transmitted from a progressor that developed AIDS to an individual who became EC (Bailey et al., 2008), thus addressing the importance of host factors in the control of infection. In particular, many investigators have searched for differences in the humoral and cellular responses between EC and natural progressors. However, humoral response, such as titer and breadth of HIV neutralizing antibody, in EC does not appear to be different when compared with that observed in natural progressors (Bailey et al., 2006a; Li et al., 2009b; Mahalanabis et al., 2009; Scheid et al., 2009), thus excluding a protective role in the early and chronic phase of infection. In support of these findings it has been reported that rituximab, a monoclonal antibody used as chemotherapeutic agent for the elimination of B cells in lymphomas, did not modify the elite status of treated HIV+ individuals, such as viral load below 50 copies/ml (Gaillard et al., 2011), although the same treatment has been reported with the reactivation of latent HBV and CMV infections (Aksoy et al., 2007) and increased viral load in chronically HIV infected (non-elite controller) individual (Huang et al., 2010).

All these findings tend to support the hypothesis that among immune responses the cell-mediated pathways might play a major role responsible for the elite status of HIV+ individuals.

## Cell-Mediated Immune Responses in Elite Controllers

### HIV and HLA

Human immunodeficiency virus infection and the viral protein Nef causes down modulation of human leukocytes antigen (HLA)-class I molecules (Schwartz et al., 1996) to avoid recognition of infected cells by cytotoxic T lymphocytes (CTL). On the other hand, natural killer (NK) cells quickly recognize infected cells by detecting down modulation of HLA-class I molecules, thus recognizing viral infected cells very soon after infection. At the same time, HLA-C and HLA-E are not down modulated upon HIV infection (Cohen et al., 1999). Thereby, HIV to avoid detection by CTL encodes mechanisms for removal of class I major histocompatibility complex (MHC) proteins from the surface of infected cells, and is protected from NK cell cytotoxicity primarily by HLA-C and HLA-E.

Well documented correlates of delayed disease progression in HIV+ individuals controlling viral load in the absence of anti-retroviral therapy include several alleles of the MHC Class I genes. HLA-B alleles have been found to be more resistant than HLA-A to the Nef-mediated downregulation (Rajapaksa et al., 2012), and few HLA-B alleles reported to contribute to the control of viral load in HIV+ individuals, as B*27, B*57, and B*14 that have been associated with efficient poly-functional CD8 response (Almeida et al., 2007a), viremia control (Fellay et al., 2007), and slow disease progression (Pereyra et al., 2010; Lazaryan et al., 2011). However, HLA allele combinations also exert a strong effect on non-progression in LTNP (McMichael and Jones, 2010; Salgado et al., 2011). Genome-wide association studies have also indicated genetic variants of MHC Class I genes (HLA-C) associated with control of viral load and disease progression through modulation of the response of CD8+ and NK cells (Fellay et al., 2007; Thomas et al., 2009), whereas others have highlighted a role of MHC Class I and III single nucleotide polymorphisms (SNPs) in determining the LTNP phenotype (Guergnon et al., 2012).

### CD8+ T lymphocytes

Human leukocytes antigen-B57 and HLA-B27 alleles have been found to be over-represented among EC (Kaslow et al., 1996; Migueles et al., 2000; Kiepiela et al., 2004; Lambotte et al., 2005), and supported by multiple studies showing that HIV-1-specific CD8+ T cells from EC were qualitatively superior (e.g., high proliferative capacity and cytotoxic activity) to those from patients with progressive disease (Migueles et al., 2002; Betts et al., 2006; Addo et al., 2007; Almeida et al., 2007a; Saez-Cirion et al., 2007). In particular, a poly-functional response to HIV stimulation was observed for CD8+ T cells isolated from EC and LTNP, and was lost in individuals becoming progressors (Betts et al., 2006; Almeida et al., 2007a; Streeck et al., 2008). Poly-functional response was characterized by efficient process of degranulation and release of perforin and granzyme B, as well as of cytokines IFN-γ, TNF-α, IL-2, and MIP-1β (Migueles et al., 2002, 2008; Hersperger et al., 2010). The increased level of poly-functional CD8+ T cells was inversely correlated with viral load (Betts et al., 2006), and findings reported in humans have been confirmed in macaques, showing that depletion of CD8+ T cells in animals controlling viral load, as the human counterpart EC and LTNP, is associated with rapid increase of viral load and drop of CD4+ T cell count (Friedrich et al., 2007). According to this set of information, it was reported that transition from LTNP to the progressor status was associated with the acquisition of viral mutations conferring escape from CD8+ T cell responses (Kemal et al., 2008), and recently confirmed in animal model (Mudd et al., 2012).

It is of interest that EC and HIV+ individuals under HAART have comparable viral loads but the presence of poly-functional CD8+ T cells remains a characteristic of EC (Dinoso et al., 2008; Ferre et al., 2009; Peris-Pertusa et al., 2010; Lopez et al., 2011), despite that therapy-treated HIV+ individuals increased the number of CD4+ T cells (Peris-Pertusa et al., 2010), meaning that this cellular population is one of the host factors responsible for the suppression of viral load rather than a consequence of the infection or exposure of CD8+ T cells to high viral loads before therapy (Lopez et al., 2011).

When CD8+ T cells from LTNP (controlling the viral load, but with detectable viral load) and progressors (unable to control viral load) were compared, the authors showed higher levels and ability to expand of CD8+ T cells when isolated from LTNP vs. progressors, although no difference between the two cohorts were observed for the functional properties of CD8+ T cell (expression of cytokines, such as MIP-1β, TNF-α, and IL-2, upon stimulation with Gag or Nef peptides) (Lopez et al., 2008). Also these findings could be interpreted as that host factors, such as CD8+ T cells, are able to control virus replication independently by exposure to low/high levels of viral load. Nonetheless HIV-specific poly-functional CD8+ T cells from EC have recently been described to be in a differentiation stage of central memory T cells (Lopez et al., 2011; Ndhlovu et al., 2012) and to be rapidly expandable upon stimulation with Gag peptides (Ndhlovu et al., 2012). Indeed, it has also been reported that the control of viral load in HIV+ in the absence of ARTis mostly associated with HLA-B27-restricted Gag-specific CD8+ T cells (having stronger avidity, poly-functionality, and clonal turnover than HLA-A restricted CD8+ T subpopulations) (Almeida et al., 2007a; Berger et al., 2011). It has recently reported that among the many factors influencing the efficacy of this protective allele in EC, expression of specific TCR clonotypes (Chen et al., 2012), and evasion from regulatory T (T_reg_) cells suppression (Elahi et al., 2011) are features associated with delayed HIV disease progression.

Recently, it has also been reported that CD8+ T cells of non-controllers or patients under HAART are characterized by loss of CD100 expression (alternative name is Sema4D, a semaphorin with important immune regulatory functions that improve antigen-specific priming by antigen presenting cells and also acts as costimulatory molecule on T cells), whereas EC maintain CD100 expression as much as non-infected individuals (Eriksson et al., 2012).

#### Mucosal immunity

Increased magnitude of CD8+ T cell response to HLA-B27 and HLA-B57-restricted Gag epitopes, such as HIV p24Gag and p17Gag as well as other non-immunodominant epitopes (Ferre et al., 2009), was reported in both blood and rectal mucosa of EC (Shacklett et al., 2003; Ibarrondo et al., 2005; Ferre et al., 2009, 2010), and increased level of poly-functional CD8+ T cells in rectal mucosa of EC (Critchfield et al., 2007, 2008; Ferre et al., 2009, 2010) has been proposed as one of the reason why these individuals experience lower depletion of CD4+ T cells than progressive individuals normally seen in this anatomical compartment of HIV progressors (Brenchley and Douek, 2008). However, CD8+ T cells in lymph nodes and gastrointestinal mucosa of infected macaques express high levels of PD-1, suggesting immune exhaustion (Velu et al., 2007), and reduced ability to clear chronic infection. These findings suggest that mucosal CD8+ T cells may be or become inadequate over time, and possibly linked to the progression from the elite to the progressor status (i.e., loss of control of viral load).

In conclusion, EC are characterized by preservation of functional CD8+ cells mounting appropriate and strong Gag-specific response, poly-functional, and at the central memory stage. Moreover, selection for mutated but less-fit viral variants, as consequent to the immune selective pressure by HLA-I/CD8+ T cells, has been proposed as an additional mechanism for the control of viral load in elite controller (Miura et al., 2009a,b,c). Indeed, HLA heterogeneity and low to undetectable HIV-specific CD8 T cell responses have been observed among HIV-1 controllers cohorts, with all the above variants together accounting for approximately 40% of all individuals with a resistant phenotype to HIV-1 disease (Emu et al., 2008; Pereyra et al., 2008). It is likely that additional genetic or immuno-virological determinants of resistance to HIV-1 disease progression are yet to be discovered (O’Brien and Nelson, 2004). In support of this interpretation is also that selection of mutated HIV variants is indicative of ongoing viral replication, and escape from T cell-mediated immunity also occurs in individuals naturally controlling HIV viral load.

### Natural killer cells

A second cellular population showing cytotoxic activity against virus infected cells is represented by NK cells, potentially contributing to viral control along with traditional adaptive immune responses in some controller individuals. Recent findings have shown association between the killer inhibitory receptor (KIR) alleles and virus control. In particular, KIR3DL1 and KIR3DS1 have been associated with delayed progression to AIDS when interacting with HLA-B alleles. Thus, NK-mediated control of HIV replication has been also in cohorts of HIV+ individuals with spontaneous control of viral load (Martin et al., 2002, 2007; Lopez-Vazquez et al., 2005; Yawata et al., 2006; Alter et al., 2007; Long et al., 2008; Kamya et al., 2011a). Of note is that HIV controllers expressing HLA-Bw4*80I on target cells and KIR3DL1 on NK cells sustain a target cell-induced NK cytotoxicity stronger that that measured for CD8+ T cells from the same individuals (Tomescu et al., 2012). Other than the interaction between HLA-B alleles and KIRs, role for HLA-C alleles in HIV-1 infection has been recently described. Genome-wide association studies have indicated that a SNP in the HLA-C promoter region as an important factor linked with virus control (Fellay et al., 2007, 2009; Thomas et al., 2009). It is not clear yet the protective mechanism of HLA-C SNP variant, but it has been proposed shown that HLA-C SNP variant is associated with increased expression of both HLA-C transcript and surface protein (Kulkarni et al., 2011), which correlates with good levels of CD4 counts, control of viral load, and slow disease progression (Thomas et al., 2009).

Several authors have also shown that HIV-1 mutation escaping from CTL responses are indeed recognized by NK cells (Alter et al., 2011; Fadda et al., 2011). However, if this situation also occurs in HIV+ individuals naturally controlling viral load, and in particular EC, will be quite difficult to assess. On the other side, it would be of interest to monitor if viral mutations influence HLA-mediated antigen presentation and recognition by CTL vs. NK cells, associated with the lack of the status of elite controller.

### CD4+ T lymphocytes

CD4+ T cells, or T helper cells (Th), play a central role in immune protection, by inducing (i) long-term maintenance of antigen-specific CD8+ memory T cells, (ii) antibody production from B cells, (iii) macrophages to develop enhanced microbicidal activity, to recruit neutrophils, eosinophils, and basophils to sites of infection and inflammation, and (iv) expression of cytokines and chemokines to orchestrate the full panoply of immune responses. Naive conventional CD4+ T cells are open to at least four distinct fates that are determined by the pattern of signals they receive during their initial interaction with the antigen; Th1, Th2, Th17, and T_reg_ cells (Zhu and Paul, 2008).

The number and functionality of CD4+ T cells constantly decrease during HIV disease progression, although progressive impairment and massive deregulation of all players of the immune system are observed in infected individuals, ultimately leading to the development of AIDS (Giorgi et al., 1999; Hellerstein et al., 1999).

In the majority of the EC population, the spontaneous control of HIV-1 infection is associated with higher levels of CD4+ T cells (average of 750 cells/ml) than viremic and therapy-treated HIV+ individuals, and slightly lower than uninfected subjects (Hunt et al., 2008; Hatano et al., 2009; Okulicz et al., 2009; Sedaghat et al., 2009; Kamya et al., 2011b). Control of the level of CD4+ T cells in EC has been ascribed to preservation of uncompromised thymic function in conjunction with extra-thymic processes that led to elevated levels of circulating recent thymic emigrants (Yang et al., 2012). However, a small proportion of EC developed declining number of CD4+ T cells despite undetectable levels of HIV-1 viremia as HIV-1 patients with progressive disease, that was associated to reduced thymic output mirrored by thymic dysfunction during untreated progressive HIV-1 infection (Hunt et al., 2008; Okulicz et al., 2009; Sedaghat et al., 2009; Yang et al., 2012).

Human immunodeficiency virus infection is characterized by uncontrolled viral replication and chronic immune activation, such as increased level of CD4+ and CD8+ T cells expressing activation markers such as CD38, HLA-DR, and/or Ki67 expression (Kestens et al., 1994; Liu et al., 1997; Benito et al., 2005; Almeida et al., 2007b; Hunt et al., 2008). Chronic immune activation is responsible for the disease progression, as clearly reported in animal models infected with simian counterpart simian immunodeficiency virus (SIV) (Silvestri et al., 2003).

Some studies report that despite having undetectable viral loads, EC had lower levels of immune activation than progressors, similar levels to that observed in HAART-suppressed individuals, although higher than uninfected subject (Hunt et al., 2008; Owen et al., 2010). On the other hand, EC have lower levels of activated HIV-specific CD4+ T cells than progressors and HAART-treated individuals (Owen et al., 2010), although maintained stronger immune response to HIV antigens than progressors, as characterized by interferon-γ (IFNγ) and IL-2 expression toward the Gag protein (Emu et al., 2005; Owen et al., 2010). The capacity to recognize minimal amounts of the immunodominant Gag293–312 peptide results from high avidity TCR/MHC interaction (Vingert et al., 2010). This capacity may play a key role in HIV control, by keeping the immune system in constant alert and allowing the induction of rapid recall responses. High levels of CD4+IL-2+INF-γ+ cell phenotype, with a large IL-2 production, is indicative of a robust Th1 response (Migueles et al., 2000; Potter et al., 2007).

Human immunodeficiency virus-specific CD4+ T cell function is partially restored by HAART-driven control of viral infection, suggesting that the characteristics of the HIV-specific CD4+ T cell response in EC, such as Gag responses, might be a consequence rather than the cause of the low viral burden (Tilton et al., 2007). Therefore, maintenance of good levels of CD4+ T cells and functional antigen-specific CD4+ T cells in EC might represent one aspect driving the potent anti-viral activity exerted by CD8+ T cells observed in this cohort of patients. This aspect is supported by experiments in the animal model of infection, such as SIV infected macaques. Experimental depletion of CD8+ T cells in macaques controlling SIV replication lead to a rapid rebound in SIV viremia, whereas the subsequent recovery of the immune system was associated with emergence of SIV-specific CD4+ and CD8+ T cell responses, thus restoring undetectable or low viral loads (Friedrich et al., 2007).

Members of the CD28 family, such as CD28 and the inhibitory molecules PD-1 and CTLA-4, contribute to the cell cycle arrest and termination of T cell activation occurring after TCR/MHC engagement. Levels of PD-1 and CTLA-4 are higher on CD4+ T cells of HIV progressors, than uninfected individuals, and correlate with persistent viremia, disease progression, and lack of cell functionality, such as low IL-2 production from HIV-specific CD4+ T cells in response to viral antigens (Day et al., 2006; Kaufmann et al., 2007). These characteristics were reported in both acute and chronic infection, either in untreated or virologically suppressed HAART-treated HIV+ individuals. On the other side, EC had very low levels of expression of CTLA-4, even lower than therapy-treated individuals (Kaufmann et al., 2007).

Few studies have addressed the question if CD4+ T cells from EC might be less susceptible to HIV infection than cells purified from HIV progressors. The results of these studies are controversial, since some authors suggested that cells from EC were resistant to infection because of selective up-regulation of cyclin-dependent kinase inhibitor p21 (Chen et al., 2011), whereas others reported resistance of EC CD4+ T cells to HIV-1 infection but showed that p21 plays no or indirect role in this phenomenon (Saez-Cirion et al., 2011). A third study reported that CD4+ T cells from EC were as susceptible to infection as CD4+ T cells from uninfected individuals (Julg et al., 2010; O’Connell et al., 2011).

Other authors searched for differences in the virion budding from infected cells (i.e., virus spreading). Indeed, it was reported that chronic progressors produced significantly more virus per infected cell than cells from EC and uninfected individuals, but budding from cells of EC and uninfected donors was similar (O’Connell et al., 2011). The hypothesis is that the higher levels of virus replication from cells of chronic progressors was a result of differences in the cellular activation level caused by high-level viremia in the patients and not an innate difference that would cause specific disease progression (O’Connell et al., 2011).

### Regulatory T cells

Regulatory T cells have been previously known as suppressor T cells, as they are a subpopulation of T lymphocytes suppressing immune responses of other cells. Thus, T_reg_ maintain tolerance to self-antigens and abrogate autoimmune disease, as mouse models have confirmed their role in autoimmune and inflammatory diseases, cancer, and organ transplantation (Dranoff, 2005; Battaglia and Roncarolo, 2011). T_regs_ are a subset of CD4+ T cells expressing CD25 and Foxp3+, and regulate immune responses by production of cytokines, including transforming growth factor-β (TGF-β), IL-10, and IL-35.

The role of this cell population during HIV infection is controversial. It has been proposed that excessive immune suppression by T_reg_ cells might be responsible for the faster progression on HIV pathogenesis (Kinter et al., 2007). On the other hand, T_reg_ cells might protect individuals from the deleterious effects of chronic immune activation that is typically observed in HIV infection (Belkaid and Rouse, 2005; Fazekas de St Groth and Landay, 2008). It has been reported that HIV individuals naturally controlling viremia, such as EC, or under effective therapy, have lower levels of T_regs_ compared with progressors, but that all of them have higher levels of T_regs_ than uninfected subjects (Brandt et al., 2011). However, other authors reported a higher levels of T_regs_ in EC than in both progressors and individuals under anti-retroviral therapy with fully suppressed infection (Owen et al., 2010), findings that were confirmed in the SIV infected macaques (Chase et al., 2007).

Reasons for those discrepancies are unclear but, at least in part, might be explained by the lack of specific T_reg_ markers, and the different ethnicity of individuals enrolled in the studies. For example, genetic expression profiling showed that ethnic background plays a role in the levels of expression of FoxP3, thus in the levels of T_reg_ cells as based on their classification based on this antigen (Torcia et al., 2008). Indeed, ethnicity of HIV+ individuals enrolled in the studies discussed above, although not reported, might be different in that the two cohorts are based in Denmark and California.

### TH17

Activity and expansion of T_reg_ are regulated by a subset of Th cells, such as IL-17 producing T helper (Th17) lymphocytes. Contrary to the T_reg_ cells, Th17 contributes to inflammation and immune responses to infectious agents, as well as to the maintenance of autoimmune diseases (Zhu and Qian, 2012). Th17 cells by the production of IL-22 stimulate epithelial cells (i.e., mucosal immunity) to produce anti-microbial proteins to clear out fungi (*Candida*) and bacteria by promoting inflammation through stimulating inflammatory cytokine release, chemokine expression, and recruitment of neutrophils (Bettelli et al., 2007; Weaver et al., 2007). Therefore lack of Th17 cells may predispose the host to opportunistic infections, clinical conditions occurring also in HIV+ individuals. Overall, Th17 cells act as counterbalance of T_reg_ cells in their ability to control/maintain peripheral tolerance and immune responses (Coimbra et al., 2012).

As to HIV infections and disease progression, it has been reported that marked and selective depletion of Th17 cells in lymphoid organs and mucosal tissue is typical of SIV disease progression, in that observed only in infected macaques developing pathogenesis but not in the natural host African green monkeys that do not develop signs of AIDS or disease progression (Favre et al., 2009). Therefore, loss of Th17 and disequilibrium with T_reg_ was associated to SIV disease progression. Findings reported in animal models have been also validated in HIV infected individuals, showing that in peripheral blood loss of Th17 cells, and imbalance of Th17/T_reg_ ratio, is characteristic of disease progression (Favre et al., 2010; Li et al., 2011). EC maintained levels of Th17 and T_reg_, as well as Th17/T_reg_ ratio, similar to that of uninfected individuals (Favre et al., 2010; Li et al., 2011). Therefore, these findings highlight the role of Th17 cells and the relevance of Th17/T_reg_ ratio imbalance.

Another important aspect to be considered is about the ability of Th17 controlling microbial infections at mucosal site, as loss of Th17 might account for the increased microbial translocation across the gastrointestinal mucosa (Brenchley et al., 2006). Therefore, strategies aimed at preventing Th17 loss will allow controlling disease progression mediated by the chronic immune activation, in part by controlling microbial translocation through gastrointestinal mucosa.

Therefore, among HIV+ individuals, EC are characterized by maintenance of good levels of CD4+ T cells (including Th17 cells), low level of activation of CD4+ T cells but strong response of CD4+ T cells to Gag antigens leading to a sustained Th1 phenotype (CD4+ T cells expressing IL-2 and IFN-γ).

## Extracellular Soluble Factors in Elite Controllers

Cytokines and chemokines are a group of low molecular weight proteins that mediate communication between immune and non-immune cells, contributing to regulate development, tissue repair, hematopoiesis, inflammation, and immune responses (Rossi and Zlotnik, 2000). They have pleiotropic activities and functional redundancy, and act in a complex network where one compound can influence the production of, and response to, many other factors. Chemotactic cytokines, chemokines, are involved in the traffic of leukocytes to lymphoid and non-lymphoid organs, as well as for the recruitment of leukocytes to injury and infection sites, in metastasis and angiogenesis (D’Souza and Harden, 1996; Rossi and Zlotnik, 2000; Gerard and Rollins, 2001; Moser and Loetscher, 2001). Apart from the chemoattractive functions, chemokines modulate inflammatory events, such as activation, costimulation, and differentiation of T cells and monocytes (Luther and Cyster, 2001; Mackay, 2001; Mellado et al., 2001).

### Cytokines and chemokines

Cytokines and chemokines play important roles in all steps of HIV life cycle, from the engagement of cellular receptors by HIV envelope for gaining entry into the target cells to the budding step (Alfano and Poli, 2001; Alfano et al., 2008). Some cytokines may limit viral spread whereas others may contribute to its propagation (Cocchi et al., 1995; Vicenzi et al., 2000; Alfano and Poli, 2005), but the cytokine effect is also dependent on the cell phenotypes. Indeed, β-chemokines inhibit HIV replication in T cells but enhances virus replication in macrophages (Schmidtmayerova et al., 1996).

*In vivo* studies have shown that EC have equal serum levels of IP-10, MCP-1, MIP-1α (Card et al., 2012), and IL-21 as uninfected individuals (Iannello et al., 2010), but higher levels of TGF-β (Card et al., 2012). On the other hand, when compared to HIV+ individuals unable to control viral load, EC showed lower levels of IP-10, MCP-1, and TGF-β, but higher levels of MIP-1α (Iannello et al., 2010; Card et al., 2012).

MIP-1α is a natural ligand for CCR5 (and CCR1), an HIV coreceptor, thus blocking HIV infection through competitive binding of CCR5 (Cocchi et al., 1995), but also inhibiting post-entry steps of HIV independent of coreceptor usage (Saunders et al., 2011). Therefore, elevated level of MIP-1-α have been associated with resistance to HIV infection and elevated level of this chemokine has been linked to delayed disease progression (Paxton et al., 1996; Saha et al., 1998; Hersperger et al., 2010). It is tempting to speculate that this chemokine might contribute to the control of viral load and disease progression in EC.

On the other hand, MCP-1 and IP-10 are known positive regulator of HIV replication and increased levels of MCP-1 have been reported to correlate with negative prognosis (Stylianou et al., 2000; Ansari et al., 2006, 2011).

As to the anti-inflammatory cytokine TGF-β, it has been reported that increased levels are associated with disease progression (Nilsson et al., 2006; Piconi et al., 2010). Therefore, in EC (vs. progressors) lower levels of TGF-β might be associated with more favorable prognosis, also due to the fact that TGF-β inhibits T cell response, and thus in EC low levels of TGF-β might allow for the strong HIV-specific CD8+ T cell response, thus control of viral load.

Interleukin-21 is mainly produced by follicular Th CD4+ cells, and exert pro-survival activity for CD4+ T cells, thus for maintenance of HIV-specific CD8+ T cells and of their cytolytic potential (Zeng et al., 2005), as well as regulator of T cells, B cells, and NK cells responses during chronic viral infections (Elsaesser et al., 2009; Frohlich et al., 2009; Yi et al., 2009; Pallikkuth et al., 2012). Indeed, IL-21 also promotes differentiation of naive CD4+ T cells into Th17 cells, which play an important role in inducing inflammation and controlling invading pathogens (Wei et al., 2007; Yang et al., 2008). Thus, IL-21 has been proposed as biomarkers of HIV disease progression but also as one of the target to be exploited for immunotherapy in HIV+ progressors.

### Defensins

Defensins are natural antibiotic peptides against bacteria and fungi (Chang and Klotman, 2004). Defensins are classified on the basis of their size and pattern of disulfide bond into α, β, and θ categories (Yang et al., 2002), and are produced constitutively or in response to microbial products or pro-inflammatory cytokines (Ganz and Lehrer, 1998; Lehrer and Ganz, 1999; Schroder, 1999). Cellular source of defensins are neutrophil, NK cells, epithelial, and dendritic cells (Agerberth et al., 2000; Chalifour et al., 2004; Rodriguez-Garcia et al., 2010), thus locating their beneficial (i.e., antibiotic) or detrimental [i.e., chronic inflammation-driven tumors, such as colorectal cancer (Albrethsen et al., 2005)] effects at mucosal site.

Alpha-defensins have also been shown to inhibit multiple steps of the HIV life cycle (Zhang et al., 2002; Chang et al., 2003; Mackewicz et al., 2003; Furci et al., 2007; Garzino-Demo, 2007), thus representing important innate defensive mechanism against HIV infection at mucosal sites, such as cervico-vaginal tissue (Cole and Cole, 2008).

It has been recently shown that immature dendritic cells from EC expressed higher level of α-defensins than progressors (Rodriguez-Garcia et al., 2009), and that the levels of α-defensins are independent of viral load, as underscored by the fact that HIV+ individuals under therapy expressed the same levels of α-defensins as progressors (Rodriguez-Garcia et al., 2009).

## Conclusion and Future Directions

Studying HIV disease and progression is not an easy task because of the high rate of mutations occurring in the viral strain, as well as to the acquired immunodeficiency, chronic immune activation, chronic inflammation, and the consequent opportunistic diseases and infections. The existence of individuals naturally controlling HIV viral load represents a unique opportunity for understanding mechanisms of the immune system patrolling viral replication and clearance of infected cells. It appears that viral genetic defects or humoral responses in EC do not play a major role controlling HIV viral load, whereas innate responses have been reported of potential interest. CTL seems to represent the main cellular phenotype controlling HIV viral load, although it must be recognized that the *in vivo* findings are resulting from a combination of cells and factors influencing each other.

Thus far the most appealing findings characteristic of EC are the strong responses mediated by CD8+ T cell and NK cells, and low levels of inflammatory markers, such as albumin, neopterin, MCP-1, and IL-10. Relevance of these findings to the prognosis of HIV disease has been clearly testify in SIV infected macaques, showing that (i) removal of CD8+ T cells in animals controlling viral load is associated with rapid increase of viral load and drop of CD4+ T cell count (Friedrich et al., 2007), whereas (ii) chronic immune activation and the consequent chronic inflammation is associated to disease progression (Chahroudi et al., 2012).

Moreover, studies in EC underscore that exposure to low level of antigen exposure is sufficient to boost and sustain strong and efficient HIV-specific immunity. However, some EC lose their ability to control viral load over time, indicating that factors other than HLA-mediated control contribute to the “elite” status. Indeed, even patients virologically suppressed on HAART for years demonstrate elevated levels of pro-inflammatory cytokines and chronic immune activation. These clinical events, associated with the extended survival time upon HAART (Fang et al., 2007), are associated with the occurring of opportunistic disease and poor outcomes (Nixon and Landay, 2010).

Indeed, HAART has been provided extreme benefits to HIV individuals, and intervening on the innate immune responses of therapy-treated individuals to restore or induce proper effector functions, as closer as possible to that of EC, is required for eradication of viral reservoir and elimination of the causes responsible for opportunistic diseases. Boosting proper innate immune responses will be required for both preventive (Li et al., 2009a) and the rapeutical strategies (Critchfield et al., 2007, 2008), but also controlling the chronic status of inflammation will be needed for reducing the occurring of opportunistic diseases, including non-AIDS associated tumors.

Levels of pro- and anti-inflammatory cytokine and chemokines are usually measured in the plasma of HIV+ individuals and considered for defining the inflammatory status as consequent to the chronic immune activation. However, plasma levels of these factors, especially those not correlated with viremia, would deserve future attention in order to unveil their functions, such as influencing activity and expansion of poly-functional CD8+ T cells in EC. Findings from clinical trials based on α1-Antitrypsin (Bristow et al., 2010, 2012) and IL-2 (Marchetti et al., 2008) have shown that strategies aimed at the manipulation of the innate immune system responses are feasible ways to enhance the control of virus life cycle (Alfano, 2010). Furthermore, it must be recognized that the net effect of a soluble factor on cellular phenotypes as well as on the control of HIV replication is depending by the interaction with all other soluble factors either in the plasma or in the extracellular milieu of organs/tissues and associated to the extracellular matrix. In other words, cell-mediated immune responses are depending on the localization of immune cells. Therefore, better comprehension of innate immune responses, and host and viral factors inducing protective immune responses in EC, particularly at the mucosal level, might reveal the proper way for the design of a protective anti-HIV vaccine.

Further studies comparing cohorts of HIV individuals such as progressors and EC, as well as in animal models, are needed but will provide invaluable insight for the prevention and control of HIV disease. In particular, studying modulation of host factors during transition from LTNP and EC to the progressor status will be of paramount relevance for establishing conditions associated to the control of viral load and immunodeficiency.

Unveiling the mechanisms associated with successful HIV control in EC will contribute to (i) better understand HIV pathogenesis, (ii) the design of therapies for the control of disease progression, and (iii) for vaccine strategies (Johnston and Fauci, 2007). In particular, because of the capacity of EC to restrict viral load below 50 copies/ml, it will be of paramount interest defining host factors and innate immune responses responsible for the (i) LTNP and EC conditions, (ii) control of viral load, and (iii) drop of CD4+ T cell counts.

## Conflict of Interest Statement

The authors declare that the research was conducted in the absence of any commercial or financial relationships that could be construed as a potential conflict of interest.
